# A Clinically Grounded Rule-Based Phenotyping Framework for Identifying Immune-Related Adverse Events in Structured Electronic Health Record Data: A Retrospective EHR Study

**DOI:** 10.21203/rs.3.rs-10233239/v1

**Published:** 2026-07-31

**Authors:** Natalya Alekhina, Kathi Mooney, Katherine Sward, Bob Wong, Wallace Akerley

**Affiliations:** University of Utah; University of Utah College of Nursing; University of Utah College of Nursing; University of Utah College of Nursing; Huntsman Cancer Institute

**Keywords:** Immune-related adverse events, Data-driven phenotyping, Electronic health records, Clinical phenotype, Immune-mediated diarrhea and colitis, Immune checkpoint inhibitors, Oncology informatics

## Abstract

**Background:**

Accurate identification of immune-related adverse events (irAEs) in structured electronic health record (EHR) data remains a major challenge due to the lack of specific diagnostic codes and inconsistent documentation. We propose a clinically grounded, generalizable framework for phenotyping irAEs in structured data, using immune-mediated diarrhea and colitis (IMDC) as a representative use case.

**Methods:**

Using a large, curated EHR-derived dataset of adults with advanced non-small cell lung cancer (NSCLC) treated with immune checkpoint inhibitors (ICIs), we developed a rule-based phenotyping framework informed by clinical practice guidelines and the clinical course of irAEs. The approach integrates (1) treatment exposure, (2) temporally anchored immunosuppressive therapy, and (3) diagnostic code patterns to identify moderate-to-severe irAEs, with IMDC used to enhance specificity.

**Results:**

We developed and demonstrated a guideline-informed framework for identifying complex treatment-related toxicities in structured EHR data, using immune-mediated diarrhea and colitis (IMDC) as a representative use case. Among 14,659 eligible patients treated with immune checkpoint inhibitors, 576 (3.93%) met criteria for potential grade 2–4 immune-related adverse events (irAEs). Of these, 58 (0.40%) were further classified as IMDC based on temporally aligned diagnostic codes. The framework demonstrated the feasibility of identifying clinically meaningful adverse events using structured data elements and guideline-informed temporal relationships.

**Conclusions:**

This study presents a scalable, clinically interpretable, and generalizable framework for phenotyping complex clinical outcomes in structured EHR data without reliance on advanced computational methods. By leveraging treatment patterns, diagnostic codes, and guideline-informed temporal relationships, the approach enhances reproducibility and portability across datasets and healthcare settings. Clinically grounded, rule-based phenotyping may serve as a practical complement to machine learning and natural language processing approaches when developing computable phenotypes for complex treatment-related toxicities.

## Background

Big, real-world, structured data offer important advantages such as sample size, diversity^1,2^, and longitudinality^2,3^, that hold the potential to answer some of the more complex oncology questions in an ethical^4^, timely^4^, and cost-efficient manner^5,6^. Yet, structured datasets pose several limitations stemming from the lack of control over how the data were collected, what variables were included, and how these variables were defined, recorded, and measured. This can result in incomplete data and data quality issues that can compromise the reliability and validity of subsequent findings^2,7^.

Immune-related adverse events (irAEs) are frequently experienced by individuals with cancer treated with immune checkpoint inhibitors^8–10^. IrAEs can affect various organs and tissues and range from mild to life-threatening in terms of severity^11,12^. Early identification of individuals at increased risk for experiencing moderate and severe irAEs can minimize treatment-related morbidity and improve patient outcomes. However, factors that predispose certain individuals to irAEs remain poorly understood. Large, longitudinal datasets hold the potential to answer this question. Yet, the absence of specific diagnostic codes or routinely captured biological markers associated with irAEs poses a significant challenge to identifying irAEs when working with structured data.

Some recently published irAE-focused studies have applied sophisticated computational techniques, such as machine learning^13^ and natural language processing^14^, to identify the outcome of interest. Nonetheless, these methods lack generalizability and may not be effective in some settings due to variability in data quality, the need for extensive computational resources, or the absence of standardization across different healthcare systems.

## Methods

To overcome these limitations, we developed and evaluated a clinically grounded, rule-based phenotyping framework for identifying irAEs in structured EHR data. Framework development consisted of three sequential steps: reviewing relevant clinical guidelines, translating guideline recommendations into clinically meaningful rule-based phenotype criteria, and refining phenotype specificity using diagnostic codes. We then evaluated the framework by identifying immune-mediated diarrhea and colitis, a common irAE^15–17^, within a large, structured dataset of individuals with advanced-stage non-small cell lung cancer (NSCLC) treated with immune checkpoint inhibitors.

### Identifying Relevant Clinical Guidelines

Well-established clinical guidelines, grounded in research and expert consensus, offer a robust foundation for identifying outcomes of interest in structured datasets. By translating guideline recommendations into clinically meaningful phenotype criteria, researchers can develop phenotyping frameworks for identifying complex clinical outcomes while maintaining clinical relevance and interpretability.

All-grade irAEs affect 10–30% of individuals^12,18,19^ treated with immune checkpoint inhibitors, with skin toxicities and IMDC being among the top two most prevalent irAEs^16,17,20–23^. The National Comprehensive Cancer Network (NCCN) classifies irAEs on a 1–4 scale in terms of severity and recommends systemic corticosteroids, such as oral prednisone or intravenous methylprednisolone at 1–2 mg/kg/day^24^, for management of grade 2–4 (moderate-severe) irAEs. Steroid therapy is indicated until irAE toxicity is reduced to grade ≤ 1, with a subsequent taper over 4–8 weeks^25,26^. ICIs are held during steroid therapy and may be resumed or permanently discontinued after the resolution of irAE^24^. Steroid-refractory irAEs often require treatment with monoclonal antibodies such as infliximab or vedolizumab^15,16,19^

### Establishing Key Temporal Relationships

Establishing key temporal relationships is pivotal when working with structured data. These relationships stem from the clinical course of the outcome of interest and include the timing of symptom onset, progression, and resolution, as well as the sequence of diagnostic tests, treatments, and follow-up visits. Understanding these temporal patterns allows researchers to accurately map the event trajectory and distinguish between cause and effect.

**Figure 1:**
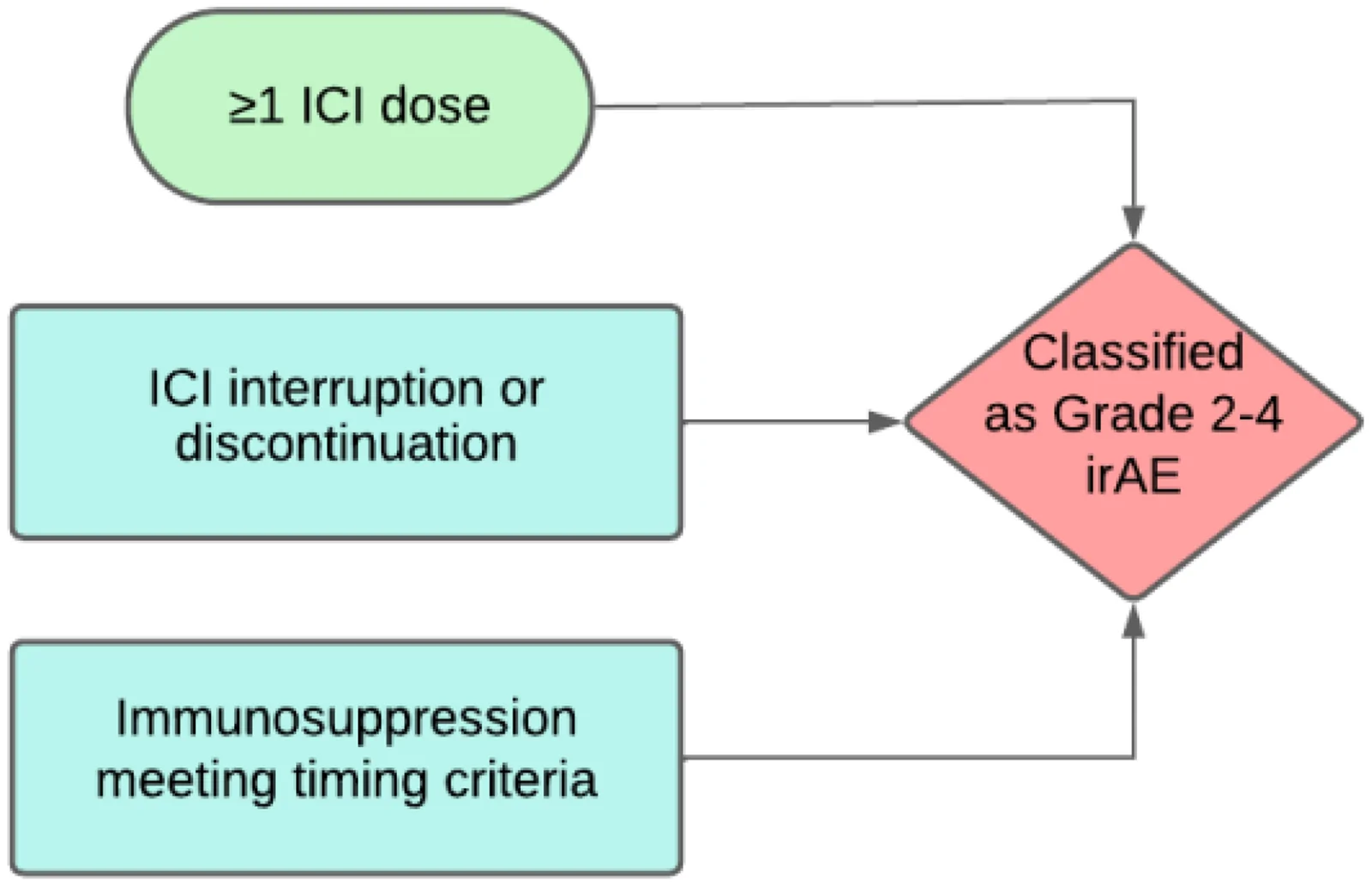
Identification of Grade 2-4 IrAEs A clinically grounded^24^, rule-based phenotyping algorithm was used to identify moderate-to-severe irAEs in structured electronic health record data. Patients were classified as having a grade 2-4 irAE if all of the following criteria were met: (1) receipt of ≥1 dose of an immune checkpoint inhibitor (ICI), (2) evidence of ICI interruption or discontinuation, and (3) receipt of immunosuppressive therapy (systemic corticosteroids, infliximab, or vedolizumab) administered either within 30 days of the last ICI dose or during a 30-90 day gap in ICI therapy.

Using NCCN guidelines for the management of irAEs^24^ and the clinical course of irAEs as a foundation, we developed a clinically grounded phenotyping algorithm (Fig. 1) that incorporates the following criteria: 1) Exposure to ≥ 1 dose of ICI 2) Evidence of immunosuppressive therapy administered within 30 days of the last ICI dose OR during a 30–90-day gap in ICI therapy. The decision to use a 30-to-90-day window was based on the average duration of steroid therapy reported in the literature^25,26^.

### Framework Evaluation

We evaluated our approach by analyzing a large, curated dataset that included de-identified, structured electronic health records of 99,628 individuals diagnosed with advanced-stage non-small cell lung cancer (NSCLC) between January 1, 2011, and May 31, 2024. This study used the US-based, electronic health record-derived deidentified Flatiron Health Research Database^27^ comprised of over 280,000 patients with NSCLC at the time of study. Patients meeting the inclusion criteria were 18 or older and received ICI therapy either as a single agent or in combination with other ICIs.

### Applying Diagnostic Codes to Enhance IrAE Characterization

In the absence of routine diagnostic markers (apart from a few laboratory abnormalities observed in patients with immune-mediated endocrinopathies, pancreatitis, or hepatitis), irAEs are challenging to discern. Diagnostic codes such as ICD-10 and ICD-9, although not specific to irAEs, can provide additional insight.

Using immune-mediated diarrhea and colitis (IMDC), a common irAE, as an example, we reviewed ICD-9 and ICD-10 diagnostic codes and their corresponding descriptions to identify gastrointestinal diagnostic categories consistent with diarrhea, colitis, and related gastrointestinal toxicities. Relevant codes and descriptions included terms such as “diarrhea,” “colitis,” “gastroenteritis,” “intestinal,” “hemorrhage,” “bowel,” “stool,” “malabsorption,” “mucosal,” “enteritis,” and “melena.” These diagnostic code categories were then applied to the previously identified irAE cohort, selecting individuals with diagnosis dates occurring within 0–90 days of the first date of immunosuppressive therapy.

## Results

A total of 14,659 patients met study eligibility criteria, with 99.92% distributed among five ICI regimens summarized in Table 1. Among eligible patients, 891 (6.08%) received oral prednisone, oral or intravenous methylprednisolone, or intravenous infliximab documented as either a prescription or an MAR. No patients treated with vedolizumab were identified.

Application of the phenotyping framework identified 576 patients who met the selection criteria for grade 2–4 irAEs and are summarized in Table 2. Among these patients, application of IMDC-specific diagnostic code categories identified 58 individuals, representing 10.07% of the irAE cohort and 0.40% of the overall study population.

## Discussion

Using a clinically grounded, guideline-informed framework, we identified potential grade 2–4 immune-related adverse events (irAEs) in 3.93% of patients treated with immune checkpoint inhibitors and immune-mediated diarrhea and colitis (IMDC) in 0.40% of the study cohort. Published estimates of moderate-to-severe irAEs among patients with NSCLC treated with pembrolizumab or nivolumab generally range from 6–14%^19,28–31^, while grade 3–4 IMDC has been reported in approximately 0.2–4%^32–34^ of patients receiving anti-PD-1 monotherapy.

While the observed incidence of IMDC fell within the lower range of published estimates, the overall incidence of moderate-to-severe irAEs was lower than previously reported. The lower incidence of irAEs in our study could be attributed to several factors. First, the proposed framework was designed to identify moderate-to-severe irAEs requiring immunosuppressive therapy and therefore would not capture milder events managed conservatively. Second, the requirement for temporally aligned treatment interruption and immunosuppressive therapy may have excluded irAEs that occurred more than 30 days after the last ICI dose. Finally, limitations inherent to structured EHR data, including incomplete documentation and historically high variability in diagnostic coding practices among clinicians,^35,36^ may have contributed to under-identification of irAEs. Additional limitations were related to data availability. Daily dose and duration information for immunosuppressive therapies were captured only within MAR and were not consistently available for self-administered medications, such as oral prednisone. As a result, corticosteroid dose and duration could not be incorporated into the phenotype definition despite their potential value for improving specificity. Opportunities to further characterize identified irAEs were also limited by the lack of routinely available clinical markers. In some settings, incorporation of laboratory values associated with specific irAEs may improve phenotype performance, while in others, review of unstructured clinical notes may be necessary for more precise classification and validation.

Despite these limitations, the proposed framework offers several advantages. By leveraging guideline-informed phenotype criteria, temporal relationships, and diagnostic codes, it provides a practical and adaptable approach for identifying clinically meaningful immune-related adverse events using routinely available structured EHR data. This approach may facilitate phenotyping in large real-world datasets and complements machine learning and natural language processing by providing a practical alternative when unstructured data or advanced computational resources are unavailable. Furthermore, the underlying approach is not limited to irAEs and may be adaptable to other treatment-related toxicities and clinical outcomes characterized by predictable management pathways, treatment-response relationships, and temporal dependencies, supporting broader application across EHR-derived datasets.

## Conclusions

This study demonstrates the feasibility of a clinically grounded, rule-based approach for identifying moderate-to-severe irAEs in structured EHR data. By leveraging guideline-informed temporal relationships and treatment patterns, this framework offers a scalable and interpretable strategy for phenotyping complex clinical outcomes using structured data alone. Although the incidence of identified irAEs was lower than reported in some prior studies, these findings underscore the importance of targeted, clinically informed frameworks for identifying clinically significant toxicities in structured datasets.

While the proposed framework holds promise, further validation against clinical notes and other data sources is needed to refine its accuracy and reliability. Future work should focus on incorporating laboratory values and unstructured clinical data, refining temporal criteria, and evaluating performance across diverse datasets and clinical settings. Additionally, collaboration with clinical teams to standardize the documentation of irAEs and related interventions may further improve data quality and support more reliable outcome identification in real-world settings.

## Figures and Tables

**Table 1.** Patient Distribution by ICI Regimen

ICI Regimen	Distinct Patient Count *n* = 14,659
Pembrolizumab	6,656 (45.41%)
Nivolumab	4,564 (31.13%)
Durvalumab	2,208 (15.06%)
Atezolizumab	656 (4.48%)
Ipilimumab, Nivolumab	563 (3.84%)
Other	12 (0.08%)

**Table 2.** Patients Meeting Grade 2-4 IrAE Selection Criteria

ICI Regimen	Prednisone *n* = 470 (81.60%)	Methylprednisolone *n* = 102 (17.71%)	Infliximab *n* = 4 (0.69%)
Pembrolizumab	202 (35.07%)	44 (7.64%)	4 (0.69%)
Nivolumab	134 (23.26%)	27 (4.69%)	0 (0.00%)
Durvalumab	87 (15.10%)	19 (3.30%)	0 (0.00%)
Atezolizumab	26 (4.51%)	3 (0.52%)	0 (0.00%)
Ipilimumab, Nivolumab	19 (3.30%)	9 (1.56%)	0 (0.00%)
Other	2 (0.35%)	0 (0.00%)	0 (0.00%)

## Data Availability

The data that support the findings of this study are the property of Flatiron Health and were used under license for the current study. Restrictions apply to the availability of these data and they are therefore not publicly available. Requests for access to the data may be submitted directly to Flatiron Health at PublicationsDataAccess@flatiron.com and are subject to review, approval, and applicable data use agreements.

## Abbreviations

IrAE: Immune-Related Adverse Event
EHR: Electronic Health Record
IMDC: Immune-Mediated Diarrhea and Colitis
NCCN: National Comprehensive Cancer Network
NSCLC: Non-Small Cell Lung Cancer
MAR: Medication Administration Record
